# Association Between Preterm-Birth Phenotypes and Differential Morbidity, Growth, and Neurodevelopment at Age 2 Years: Results From the INTERBIO-21st Newborn Study

**DOI:** 10.1001/jamapediatrics.2020.6087

**Published:** 2021-03-01

**Authors:** Jose Villar, María C. Restrepo-Méndez, Rose McGready, Fernando C. Barros, Cesar G. Victora, Shama Munim, Aris T. Papageorghiou, Roseline Ochieng, Rachel Craik, Hellen C. Barsosio, James A. Berkley, Maria Carvalho, Michelle Fernandes, Leila Cheikh Ismail, Ann Lambert, Shane A. Norris, Eric O. Ohuma, Alan Stein, Chrystelle O. O. Tshivuila-Matala, Krina T. Zondervan, Adele Winsey, Francois Nosten, Ricardo Uauy, Zulfiqar A. Bhutta, Stephen H. Kennedy

**Affiliations:** 1 Nuffield Department of Women's & Reproductive Health, University of Oxford, Oxford, United Kingdom; 2 Oxford Maternal and Perinatal Health Institute, Green Templeton College, University of Oxford, Oxford, United Kingdom; 3 Shoklo Malaria Research Unit, Mahidol-Oxford Tropical Medicine Research Unit, Faculty of Tropical Medicine, Mahidol University, Mae Sot, Thailand; 4 Centre for Tropical Medicine and Global Health, University of Oxford, Oxford, United Kingdom; 5 Programa de Pós-Graduação em Saúde e Comportamento, Universidade Católica de Pelotas, Pelotas, Brazil; 6 Programa de Pós-Graduação em Epidemiologia, Universidade Federal de Pelotas, Pelotas, Brazil; 7 Division of Women and Child Health, Department of Obstetrics and Gynaecology, Aga Khan University, Karachi, Pakistan; 8 Faculty of Health Sciences, Aga Khan University, Nairobi, Kenya; 9 KEMRI Coast Centre for Geographical Medicine and Research, University of Oxford, Kilifi, Kenya; 10 KEMRI Centre for Global Health Research, Liverpool School of Tropical Medicine, Liverpool, United Kingdom; 11 KEMRI Wellcome Trust Research Programme, Nairobi, Kenya; 12 Faculty of Medicine, Department of Paediatrics, University of Southampton, Southampton, United Kingdom; 13 Clinical Nutrition and Dietetics Department, University of Sharjah, Sharjah, United Arab Emirates; 14 SAMRC Developmental Pathways For Health Research Unit, Department of Paediatrics and Child Health, University of the Witwatersrand, Johannesburg, South Africa; 15 Maternal, Adolescent, Reproductive and Child Health Centre, London School of Hygiene and Tropical Medicine, London, United Kingdom; 16 Department of Psychiatry, University of Oxford, Oxford, United Kingdom; 17 MRC/Wits Rural Public Health and Health Transitions Research Unit (Agincourt), School of Public Health, Faculty of Health Sciences, University of the Witwatersrand, Johannesburg, South Africa; 18 Health, Nutrition and Population Global Practice, World Bank Group, Washington, DC; 19 Wellcome Centre for Human Genetics, University of Oxford, Oxford, United Kingdom; 20 Department of Nutrition and Public Health Interventions Research, London School of Hygiene and Tropical Medicine, London, United Kingdom; 21 Center for Global Child Health, Hospital for Sick Children, Toronto, Ontario, Canada

## Abstract

**Importance:**

The etiologic complexities of preterm birth remain inadequately understood, which may impede the development of better preventative and treatment measures.

**Objective:**

To examine the association between specific preterm-birth phenotypes and clinical, growth, and neurodevelopmental differences among preterm newborns compared with term newborns up to age 2 years.

**Design, Setting, and Participants:**

The INTERBIO-21st study included a cohort of preterm and term newborn singletons enrolled between March 2012 and June 2018 from maternity hospitals in 6 countries worldwide who were followed up from birth to age 2 years. All pregnancies were dated by ultrasonography. Data were analyzed from November 2019 to October 2020.

**Exposures/Interventions:**

Preterm-birth phenotypes.

**Main Outcomes and Measures:**

Infant size, health, nutrition, and World Health Organization motor development milestones assessed at ages 1 and 2 years; neurodevelopment evaluated at age 2 years using the INTERGROWTH-21st Neurodevelopment Assessment (INTER-NDA) tool.

**Results:**

A total of 6529 infants (3312 boys [50.7%]) were included in the analysis. Of those, 1381 were preterm births (mean [SD] gestational age at birth, 34.4 [0.1] weeks; 5148 were term births (mean [SD] gestational age at birth, 39.4 [0] weeks). Among 1381 preterm newborns, 8 phenotypes were identified: no main maternal, fetal, or placental condition detected (485 infants [35.1%]); infections (289 infants [20.9%]); preeclampsia (162 infants [11.7%]); fetal distress (131 infants [9.5%]); intrauterine growth restriction (110 infants [8.0%]); severe maternal disease (85 infants [6.2%]); bleeding (71 infants [5.1%]); and congenital anomaly (48 infants [3.5%]). For all phenotypes, a previous preterm birth was a risk factor for recurrence. Each phenotype displayed differences in neonatal morbidity and infant outcomes. For example, infants with the no main condition detected phenotype had low neonatal morbidity but increased morbidity and hospitalization incidence at age 1 year (odds ratio [OR], 2.2; 95% CI, 1.8-2.7). Compared with term newborns, the highest risk of scoring lower than the 10th centile of INTER-NDA normative values was observed in the fine motor development domain among newborns with the fetal distress (OR, 10.6; 95% CI, 5.1-22.2) phenotype.

**Conclusions and Relevance:**

Results of this study suggest that phenotypic classification may provide a better understanding of the etiologic factors and mechanisms associated with preterm birth than continuing to consider it an exclusively time-based entity.

## Key Points

**Question:**

Are specific phenotypes in preterm newborns associated with clinical, growth, and neurodevelopmental differences at age 2 years compared with term newborns?

**Findings:**

In this cohort study of 6529 preterm and term newborns who were followed up from birth to age 2 years, 8 preterm-birth phenotypes were identified: no main maternal, fetal, or placental condition detected (35%); infections (21%); preeclampsia (12%); fetal distress (10%); intrauterine growth restriction (8%); severe maternal disease (6%); bleeding (5%); and congenital anomaly (4%). Each phenotype was associated with substantial differences in neonatal morbidity and infant outcomes.

**Meaning:**

The study’s findings support the use of phenotypic classification for preterm births.

## Teaser

This cohort study, part of the INTERBIO-21st Newborn Study, an extension of the INTERGROWTH-21st Project, examines the association between preterm-birth phenotypes and clinical, growth, and neurodevelopmental differences among preterm newborns compared with term newborns up to age 2 years.

## Introduction

Preterm birth is a heterogeneous syndrome, which has been previously described in terms of 3 factors: spontaneous or medically induced labor, presence or absence of preterm premature rupture of membranes, and mode of delivery.^1^ A phenotypic classification system, incorporating etiologically associated maternal, fetal, and placental characteristics as well as signs of parturition and pathway to delivery, has also been proposed.^2,3,4^ Twelve preterm-birth phenotypes (ie, separate biological entities with specific risk factors, newborn anthropometry, and risk of neonatal morbidity and mortality)^5^ have previously been identified. The present INTERBIO-21st Newborn Study^6^ comprised phase 2 of the International Fetal and Newborn Growth Consortium for the 21st Century (INTERGROWTH-21st) Project, a population-based research initiative involving almost 70 000 mothers and infants worldwide that was conducted from 2008 to 2015. In this study, we investigated whether preterm-birth phenotypes were associated with clinical, epidemiological, growth, and neurodevelopmental differences among preterm and term infants up to age 2 years.

## Methods

The INTERBIO-21st Newborn Study included a cohort of preterm and term newborns who were enrolled from March 2012 to June 2018 and followed up from birth to age 2 years. The study was approved by the Oxfordshire Research Ethics Committee C, institutional research ethics committees at participating sites, and corresponding regional authorities. All mothers provided written informed consent. This study followed the Strengthening the Reporting of Observational Studies in Epidemiology (STROBE) reporting guideline for cohort studies.

### Study Sites and Participants

The study was conducted in Pelotas, Brazil; Nairobi, Kenya; Kilifi, Kenya; Karachi, Pakistan; Soweto, South Africa; Mae Sot, Thailand; and Oxford, United Kingdom. The sites and study populations have been described elsewhere.^6^

In brief, the study enrolled preterm newborns who were live born at 23 weeks 0 days’ to 36 weeks 6 days’ gestation and term newborns who were live born at 37 weeks 0 days’ to 41 weeks 6 days’ gestation.^7^ All newborns were naturally conceived singletons with mothers who were age 18 years and older and residing in the catchment area of the participating hospital. Gestational age was estimated by ultrasonographic measurement of crown-rump length at less than 14 weeks 0 days’ gestation (75% of newborns) or head circumference at less than 24 weeks 0 days’ gestation (25% of newborns) using INTERGROWTH-21st standards.^8,9^ Given the design of the INTERBIO-21st study, we selected a higher proportion of newborns with lower birth weight (ie, < third centile).

Deliveries were screened daily using a tablet computer (iPad; Apple),^6^ with software oversampling at lower gestational ages to increase statistical power for studying the highest-risk subgroups by producing a higher proportion of exposures and adverse neonatal outcomes. To correct for the oversampling of newborns who were small for gestational age (defined as birth weight <10th centile based on INTERGROWTH-21st standards) in the present analysis, we generated a term newborn group consisting of term infants who were appropriately grown for gestational age (defined as birth weight ≥10th centile based on INTERGROWTH-21st standards); approximately 10% of the term newborn group included randomly selected term infants who were small for gestational age to reflect the incidence of newborns who are small for gestational age in the general population.

### Neonatal and Child Outcomes

The anthropometric measurement protocols, training materials, and quality control procedures were based on the World Health Organization (WHO) Multicentre Growth Reference Study,^10^ which produced the WHO Child Growth Standards.^11^ In brief, newborn measures were obtained within 12 hours of birth (and no later than 24 hours after birth) using identical equipment at all sites. An electronic scale (sensitivity, 10-20 g; Seca) was used to measure birth weight, and an infantometer (Harpenden; Chasmors) was used to measure recumbent length.^10^ Head circumference was measured using metallic nonextendable tape (Chasmors). The anthropometrists independently took all measures twice and compared values using maximum allowable differences of 50 g for weight, 7 mm for length, and 4 mm for head circumference. If any difference exceeded those values, both observers independently performed the relevant measurement a second time and, if necessary, a third time.^12,13^ The Oxford Anthropometric Standardization Unit regularly monitored staff performance and recommended retraining if measurements consistently exceeded the maximum allowable differences.

Standardized care and feeding practices were implemented using INTERGROWTH-21st protocols. Exclusive breastfeeding until age 6 months was encouraged, with standard supplementation for preterm newborns.^14^

Each mother provided detailed information about the infant’s health and severe morbidities at birth, age 1 year, and age 2 years using project-specific forms.^15^ We used an unweighted composite neonatal outcome score comprising a severe neonatal morbidity index that included 1 or more of the following severe neonatal complications: bronchopulmonary dysplasia, hypoxic-ischemic encephalopathy, sepsis, anemia (requiring transfusion), periventricular hemorrhage or leukomalacia, retinopathy, necrotizing enterocolitis (Bell stage 2 or higher), and patent ductus arteriosus (requiring intervention).^1,16,17,18^ At ages 1 and 2 years, we assessed (1) hospital admission (≥1 admission); (2) diagnosis and treatment for infections requiring antibiotic medication (≥3 regimens), otitis media, pneumonia, bronchiolitis, parasitosis, diarrhea, vomiting, exanthema, skin disease, fever lasting 3 or more days (≥3 episodes), and meningitis; (3) neurologic disorders, including seizures and cerebral palsy; and (4) severe clinical conditions, including cardiovascular problems, gastroesophageal reflux, hemolytic conditions, trauma from injury, or any condition requiring surgery.

At birth, we measured weight, length, and head circumference following INTERGROWTH-21st protocols. At ages 1 and 2 years, we measured weight, length, and head circumference following WHO protocols.^10^ Motor development was assessed using the chronological age of the child by comparing parental information with 4 WHO developmental milestones: sitting without support, crawling on hands and knees, standing alone, and walking alone.^19^

We assessed neurodevelopment at age 2 years using the INTERGROWTH-21st Neurodevelopment Assessment (INTER-NDA), which measures multiple dimensions of early development among children aged 22 to 30 months.^20^ The INTER-NDA is implemented by nonspecialists^21^ and uses mixed methods to measure cognition, language development, fine and gross motor skills, and positive and negative behavior. The tool has been validated against the Bayley Scales of Infant Development^21,22^ and has indicated good interrater and test-retest reliability.^20^ Vision was assessed using the Cardiff Visual Acuity and Contrast Sensitivity tests.^23^ We estimated the proportion of children scoring lower than the 10th centile on the INTER-NDA and on visual tests using international standards.^24^

### Statistical Analysis

To construct the preterm-birth phenotypes (eTable 1 in the Supplement), we were guided by the work of the INTERGROWTH-21st Consortium, which provided a conceptual frame and etiologic factors^2,3,4^ that were empirically tested.^5^ The same definitions of maternal, fetal, and placental conditions and the same analytical strategy using a 2-step cluster analysis (via the 2-step cluster algorithm in SPSS software, version 25; SPSS Statistics) were then applied to the data collected for the present study.^5^ This approach allowed us to compare the distribution of clusters and their proportional contribution across populations.

Quality was assessed based on silhouette measures of cohesion and separation. Clustering was considered satisfactory if the silhouette statistic was 0.6 or higher (range, −1.0 to 1.0).^25^ After the first analyses, we merged clusters associated with infections (extrauterine infection, chorioamnionitis, and perinatal sepsis) because each infection had low statistical power when analyzed separately (eTable 1 in the Supplement). Of 44 fetal anemia cases, cluster analysis categorized 28 cases (63.6%) as perinatal sepsis, 6 cases (13.6%) as mid to late pregnancy, 5 cases (11.4%) as fetal distress, 3 cases (6.8%) as preeclampsia, and 2 cases (4.5%) as congenital anomaly. Because most cases were associated with perinatal sepsis, we included those newborns in the infections group.

To assess associations between maternal risk factors and phenotypes, we performed multinomial logistic regression analyses to model nominal outcome variables. Odds ratios (ORs) for each phenotype (and for all preterm newborns) compared with the term newborn group were estimated based on multinomial modeling. Model 1 was adjusted for maternal age, height, first trimester and prepregnancy body mass index (calculated as weight in kilograms divided by height in meters squared), years of education, presence of prepregnancy mental illness, length of menstrual cycle, and smoking during pregnancy. Model 2 was adjusted for all variables included in model 1 plus the number of previous pregnancies, miscarriages, and terminations. Model 3 was adjusted for all variables included in model 2 plus the number of previous births, low birth weight (defined as <2500 g) and preterm infants, and neonatal deaths. We found no data that violated the independence of irrelevant alternatives assumption in our models.

For all newborns, we generated age- and sex-specific *z* scores for weight, length, head circumference, weight for length, and percentage of newborns lower than the 10th centile compared with INTERGROWTH-21st standards^7^ and preterm reference charts.^26^ At ages 1 and 2 years, similar *z* scores and centiles for weight, length, and head circumference were generated compared with the WHO Child Growth Standards.^11^ We used 1-way analysis of variance to test whether the means of each *z* score were different across the phenotypes and the term newborn group. Phenotypic analyses at age 2 years used chronological rather than corrected age.^27^

We performed logistic regression analysis to assess associations between phenotypes and neonatal, 1-year, and 2-year morbidity indices, presented as ORs with 95% CIs adjusted for study site. For the neonatal morbidity analyses, we compared individual preterm-birth phenotypes with the phenotype for no main maternal, fetal, or placental condition detected. Unadjusted and adjusted analyses were conducted for gestational age. Robust SEs were estimated in all logistic association models using the vce (cluster clustvar) package in Stata software, version 15 (StataCorp).

We used a Kruskal-Wallis nonparametric test to examine the equality of age distribution at the achievement of gross motor development milestones among phenotypes and the term newborn group. All analyses were performed using Stata software, version 15. Data were analyzed from November 2019 to October 2020.

## Results

We prospectively enrolled 7540 women between 2012 and 2019; of those, 202 women were lost to follow-up or withdrew consent. After excluding 27 infants with missing birth weights, 7311 newborns (1381 preterm newborns and 5930 term newborns; 4633 newborns who were appropriately grown for gestational age and 1297 newborns who were small for gestational age) remained (eFigure 1 in the Supplement). To restore the prevalence of infants who were small for gestational age to 10%, we randomly excluded 782 term newborns who were small for gestational age from the term newborn group. After all exclusions, a total of 6529 infants (3312 boys [50.7%]) were included in the final analysis. Of those, 1381 newborns were preterm births (mean [SD] gestational age at birth, 34.4 [0.1] weeks; mean [SD] maternal age, 29.3 [0.2] years) and 5148 newborns were term births (mean [SD] gestational age at birth, 39.4 [0] weeks; mean [SD] maternal age, 29.3 [0.1] years). The overall follow-up rate was 78% (71% for preterm newborns and 80% for term newborns) at 1 year and 67% (64% for preterm newborns and 68% for term newborns) at 2 years (eFigure 1 in the Supplement).

Each site’s contribution to the total study population ranged from 7.6% (Karachi, Pakistan) to 25.0% (Oxford, United Kingdom). For the preterm group, each site’s contribution ranged from 6.7% (Soweto, South Africa) to 27.0% (Oxford, United Kingdom). Sociodemographic characteristics were similar across groups from different sites.

Among 1381 preterm newborns, the largest phenotype was no main maternal, fetal, or placental condition detected (no main condition phenotype; 485 infants [35.1%]). Cluster selection also derived phenotypes with 1 main condition (with the exception of the severe maternal disease phenotype, comprising 85 infants [6.2%]), indicating that preterm birth was a heterogeneous syndrome. These phenotypes comprised infections (289 infants [20.9%]), preeclampsia (162 infants [11.7%]), fetal distress (131 infants [9.5%]), intrauterine growth restriction (110 infants [8.0%]), bleeding (71 infants [5.1%]), and congenital anomaly (48 infants [3.5%]) (eTable 3 in the Supplement). The main congenital anomalies diagnosed were in the face, heart, and limbs, with no observable common factor.

Cesarean deliveries were more common among preterm newborns compared with term newborns (696 of 1381 infants [50.4%] vs 1778 of 5148 infants [34.5%], respectively) and among preterm newborns with specific phenotypes, such as preeclampsia (124 of 162 infants [76.5%]), bleeding (61 of 71 infants [85.9%]), fetal distress (123 of 131 infants [93.9%]), and severe maternal disease (55 of 85 infants [64.7%]) (eTable 2 in the Supplement). Conditions such as preterm premature rupture of membranes were more common among those with preterm-birth phenotypes for infections (174 of 289 infants [60.2%]), severe maternal disease (32 of 85 infants [37.6%]), and congenital anomaly (17 of 48 infants [35.4%]). In addition, newborns with preterm phenotypes had lower birth weights (mean [SD], 2210 [15.9] g vs 3200 [6.2] g), shorter length at birth (mean [SD], 44.6 [0.1] cm vs 48.9 [0] cm), smaller head circumference at birth (mean [SD], 31.3 [0.1] cm vs 33.9 [0] cm), higher neonatal morbidity (eg, hospitalization in neonatal intensive care unit for >1 day, 755 of 1381 infants [54.7%] vs 380 of 5148 infants [7.4%]), and higher neonatal mortality (55 of 1381 infants [4.0%] vs 8 of 5148 infants [0.2%]) compared with the term newborn group.

For all preterm-birth phenotypes, a previous preterm birth was a risk factor for recurrence of preterm delivery (results for each risk factor assessed in each model are available in eFigure 2 in the Supplement). Associations with preterm phenotypes were also identified, indicating a differential risk according to preterm phenotype. The no main condition phenotype was associated with young maternal age (OR, 1.6; 95% CI, 1.2-2.2), nulliparity (OR, 2.0; 95% CI, 1.3-3.1), short maternal stature (OR, 1.4; 95% CI, 1.1-1.8), fewer maternal years of education (OR, 1.5; 95% CI, 1.1-1.9), and 2 or more previous miscarriages (OR, 1.5; 95% CI, 1.1-2.2). The preeclampsia phenotype was associated with low maternal body mass index (OR, 2.3; 95% CI, 1.0-4.9) or overweight maternal status (OR, 3.0; 95% CI, 2.1-4.5), nulligravidity (OR, 1.9; 95% CI, 1.3-3.0), and delivery of a previous infant with low birth weight (OR, 2.8; 95% CI, 1.5-5.3). The infections phenotype was associated with both younger (OR, 1.9; 95% CI, 1.3-2.9) and older (OR, 1.4; 95% CI, 1.1-1.9) maternal age and previous termination of pregnancy (OR, 1.7; 95% CI, 1.1-2.8). The infections phenotype was associated with both younger (OR, 1.9; 95% CI, 1.3-2.9) and older (OR, 1.4; 95% CI, 1.1-1.9) maternal age and previous termination of pregnancy (OR, 1.7; 95% CI 1.1-2.8). The intrauterine growth restriction phenotype was associated with short maternal stature (OR, 2.2; 95% CI, 1.3-3.8), smoking during pregnancy (OR, 5.0; 95% CI, 2.9-8.6), and previous miscarriages (OR, 2.2; 95% CI, 1.2-3.9).

### Newborn Size and Postnatal Growth

Overall, 249 preterm newborns (18.0%) were small for gestational age and most were associated with the intrauterine growth restriction (73 infants [66.0%]), preeclampsia (42 infants [26.1%]), and fetal distress (33 infants [24.9%]) phenotypes (eFigure 3 in the Supplement).

**Figure 1.**
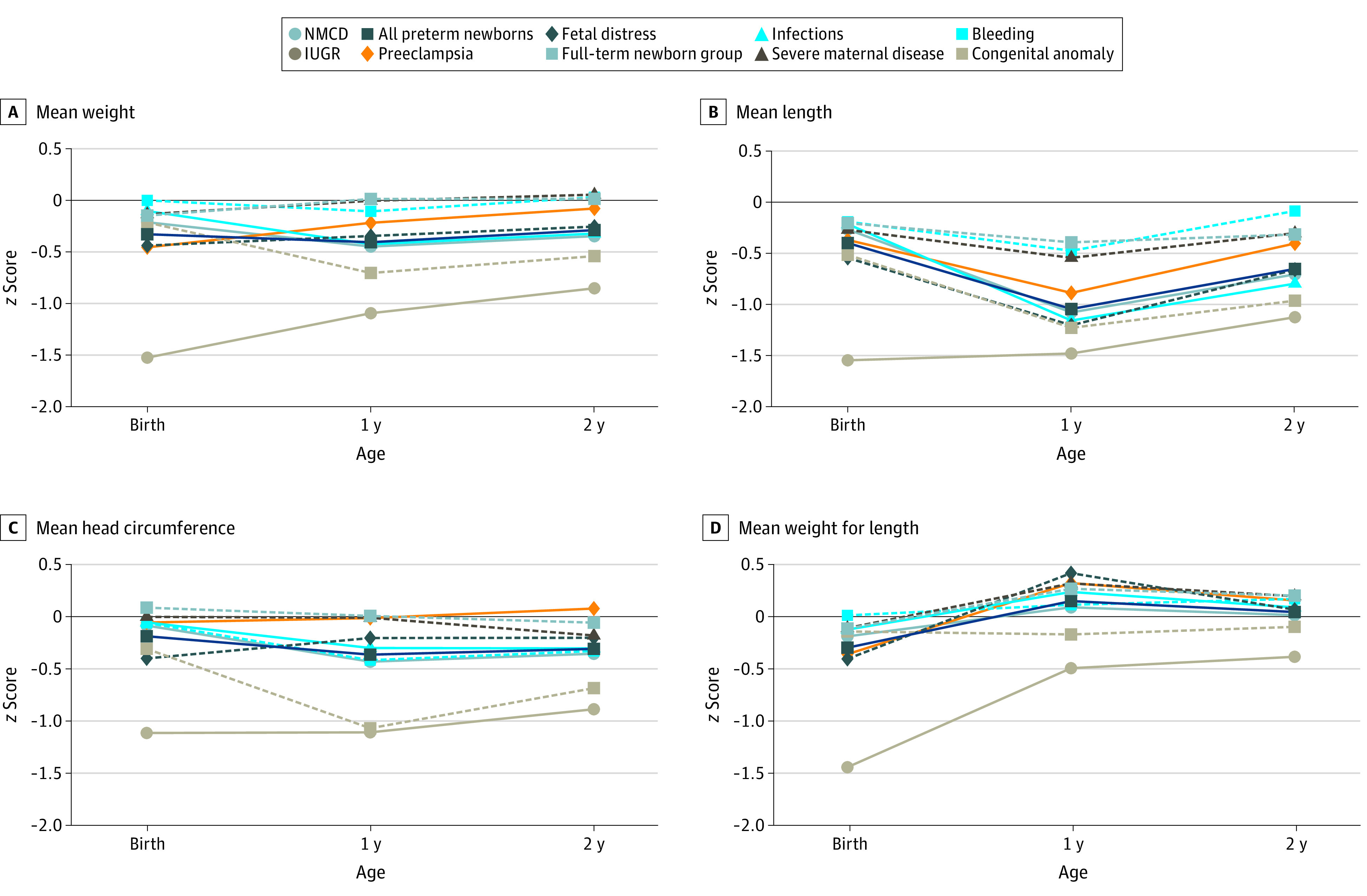
Mean *z* Scores at Birth and Ages 1 and 2 Years According to Preterm-Birth Phenotype A-D, Mean values. Gestational age- and sex-specific centiles at birth were compared with the international INTERGROWTH-21st newborn size standards and reference charts for very preterm size at birth.^7,25^ Age- and sex-specific centiles at ages 1 and 2 years were compared with the international World Health Organization Child Growth Standards.^26^ *P* < .001 for all comparisons based on analysis of variance. IUGR indicates intrauterine growth restriction; NMCD, no main condition detected.

Figure 1 presents anthropometric patterns from birth to age 2 years according to phenotype and for all preterm newborns and the term newborn group, expressed as mean *z* scores (actual *z* score values are presented in eTable 4 in the Supplement). Using *z* scores from birth to childhood was achievable because international standards, constructed using the prescriptive approach from the WHO,^28^ were available throughout the childhood growth period. Figure 1A shows birth weight distributed within 0 and −0.5 SD of the standards, with only the intrauterine growth restriction phenotype substantially lower than −1.0 SD. Beginning at age 1 year, 3 growth patterns emerged. The first pattern, which was close to the WHO Child Growth Standards 50th centile, consisted of the term newborn group and preterm newborns with the bleeding, preeclampsia, and severe maternal disease phenotypes. The second pattern consisted of all preterm newborns plus those with the no main condition, infections, and fetal distress phenotypes. The third pattern comprised newborns with the intrauterine growth restriction phenotype, which indicated a pattern that was increasing but remained lower than −1.0 SD of the WHO Child Growth Standards.^11^

Figure 1B presents the marked decrease in *z* scores for length and height at age 1 year, with a mixed pattern at age 2 years. Height at age 2 years appeared less affected in the term newborn group and in preterm newborns with some phenotypes. Newborns with the bleeding phenotype remained close to the 50th centile; those with the congenital anomaly and intrauterine growth restriction phenotypes were close to −1.0 SD of the WHO Child Growth Standards.^11^ Figure 1C shows a parallel pattern for postnatal head circumference growth among phenotypes. By age 2 years, different gradients were observed from newborns with the preeclampsia phenotype to the term newborn group and preterm newborns with other phenotypes, including the no main condition phenotype. Figure 1D shows patterns for birth weight, birth length, and postnatal body mass index, expressed as *z* scores. Unlike the 3 patterns in Figure 1A-C, similar trajectories were observed for all groups higher than the 50th centile (ie, children started being overweight for their height by age 1 year).

### Neonatal and Child Morbidity

**Figure 2.**
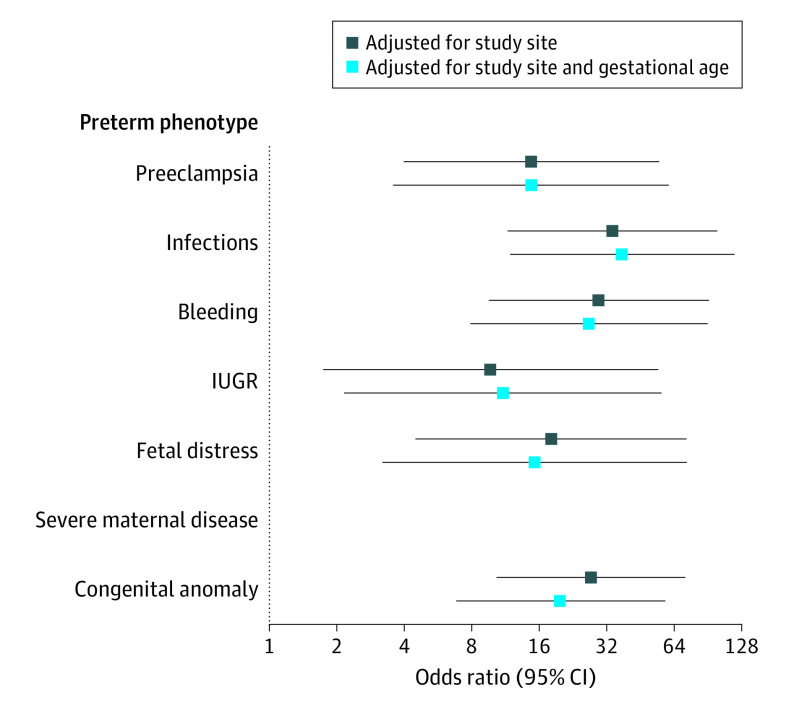
Severe Neonatal Morbidity Index According to Preterm-Birth Phenotype The Severe Neonatal Morbidity Index includes bronchopulmonary dysplasia, hypoxic-ischemic encephalopathy, sepsis, neonatal anemia (requiring transfusion), periventricular hemorrhage or leukomalacia, retinopathy of prematurity, necrotizing enterocolitis (Bell stage 2 or higher), and patent ductus arteriosus (requiring pharmacologic treatment or surgery). There were no cases of severe neonatal morbidity in newborns with the severe maternal disease phenotype. Odds ratios are based on comparisons with the NMCD phenotype. The 95% CIs were based on robust SEs. IUGR indicates intrauterine growth restriction; NMCD, no main condition detected.

Figure 2 shows ORs for neonatal severe morbidity by preterm-birth phenotype compared with the no main condition phenotype. After adjusting for study site, all preterm-birth phenotypes had an increased risk of severe neonatal morbidity compared with the no main condition phenotype. The results remained similar after adjustment for gestational age.

An analysis of each phenotype separately revealed considerable heterogeneity. The highest risk of severe neonatal morbidity was found in newborns with the infections (OR, 33.9; 95% CI, 11.6-98.9), bleeding (OR, 29.4; 95% CI, 9.6-90.6), and congenital anomaly (OR, 27.1; 95% CI, 10.4-71.4) phenotypes. The lowest risk of severe neonatal morbidity was observed in newborns with the intrauterine growth restriction phenotype (OR, 9.7; 95% CI, 1.7-53.9); however, newborns with this phenotype had the highest risk of neonatal and postnatal growth restriction (Figure 1). There was minimal to no attenuation in ORs for preterm-birth phenotypes compared with the no main condition phenotype when adjusting for gestational age (eg, for the infections phenotype, OR, 37.5 [95% CI, 11.9-118.2]; for the bleeding phenotype, OR, 26.6 [95% CI, 7.8-90.7]; and for the congenital anomaly phenotype, OR, 19.9 [95% CI, 6.8-58.1]), indicating that underlying conditions were associated with increased morbidity independent of gestational age (Figure 2).

The phenotypes associated with different morbidities at age 1 year among preterm newborns compared with the term newborn group are shown in eTable 5 in the Supplement. Excluding the congenital anomaly phenotype, newborns with the no main condition (OR, 2.2; 95% CI, 1.8-2.7), infections (OR, 3.4; 95% CI, 2.3-5.2), and fetal distress (OR, 3.7; 95% CI, 2.4-5.9) phenotypes had a higher risk of hospitalization. Those with the preeclampsia (OR, 1.9; 95% CI, 1.5-2.6) and intrauterine growth restriction (OR, 2.0; 95% CI, 1.2-3.3) phenotypes had more infection-associated episodes. Newborns with the no main condition (OR, 4.6; 95% CI, 2.1-9.8), bleeding (OR, 3.5; 95% CI, 1.2-10.2), intrauterine growth restriction (OR, 2.8; 95% CI, 1.0-7.6), and fetal distress (OR, 4.4; 95% CI, 1.0-18.9) phenotypes had a higher risk of neurologic disorders, and those with the infections (OR, 1.8; 95% CI, 1.3-2.4), bleeding (OR, 2.3; 95% CI, 1.2-4.3), and fetal distress (OR, 2.2; 95% CI, 1.7-2.8) phenotypes had a higher risk of severe clinical conditions.

Table 1 shows morbidity data at age 2 years. For infection-associated episodes, the highest-risk phenotypes were fetal distress (OR, 1.8; 95% CI, 1.0-3.2), preeclampsia (OR, 1.5; 95% CI, 0.8-2.6), no main condition (OR, 1.4; 95% CI, 1.2-1.6), and severe maternal disease (OR, 1.4; 95% CI, 1.0-2.2). For neurologic disorders, the highest-risk phenotypes were preeclampsia (OR, 6.9; 95% CI, 2.7-17.6) and fetal distress (OR, 3.0; 95% CI, 1.0-9.1). For severe clinical conditions, the highest-risk phenotype was severe maternal disease (OR, 1.9; 95% CI, 1.2-3.1). As with newborn size and postnatal growth, considering preterm birth as a single entity would have obscured the different morbidity patterns for each phenotype. Notably, there was an increased risk of infections at age 2 years among preterm infants with the no main condition phenotype, a group that is considered to have a low risk of adverse health outcomes in childhood.

**Table 1.** Morbidity Among Children at Age 2 Years According to Preterm-Birth Phenotype^a^

Variable	Children with ≥1 hospitalization		Children diagnosed with or treated for ≥3 infections		Children diagnosed with or treated for neurologic disorders		Children diagnosed with or treated for severe clinical conditions	
	No./total No. (%)	OR (95% CI)	No/total No. (%)^b^	OR (95% CI)^b^	No./total No. (%)^c^	OR (95% CI)^c^	No./total No. (%)^d^	OR (95% CI)^d^
Phenotype								
No main condition detected	40/287 (13.9)	1.4 (1.1-1.8)	47/287 (16.4)	1.4 (1.2-1.6)	7/287 (2.4)	2.2 (0.8-5.8)	25/287 (8.7)	0.8 (0.5-1.3)
Preeclampsia	9/92 (9.8)	1.1 (0.3-3.6)	17/92 (18.5)	1.5 (0.8-2.6)	7/92 (7.6)	6.9 (2.7-17.6)	11/92 (12.0)	1.1 (0.6-1.9)
Infections	25/150 (16.7)	1.6 (1.2-2.0)	16/150 (10.7)	1.1 (0.4-3.0)	4/150 (2.7)	2.4 (0.7-8.5)	13/150 (8.7)	0.8 (0.5-1.1)
Bleeding	9/44 (20.5)	2.3 (1.5-3.4)	6/44 (13.6)	1.4 (0.4-4.4)	0/44 (0)	NA	2/44 (4.5)	0.4 (0.2-0.8)
Intrauterine growth restriction	12/47 (25.5)	3.1 (1.6-6.0)	4/47 (8.5)	0.9 (0.2-3.3)	1/47 (2.1)	1.7 (0.2-13.3)	7/47 (14.9)	1.6 (0.5-5.1)
Fetal distress	17/76 (22.4)	2.7 (1.6-4.5)	12/76 (15.8)	1.8 (1.0-3.2)	3/76 (3.9)	3.0 (1.0-9.1)	10/76 (13.2)	1.5 (0.9-2.6)
Severe maternal disease	8/55 (14.5)	1.7 (0.8-3.6)	10/55 (18.2)	1.4 (1.0-2.2)	1/55 (1.8)	1.4 (0.4-5.3)	11/55 (20.0)	1.9 (1.2-3.1)
Congenital anomaly	7/29 (24.1)	2.5 (1.0-6.2)	3/29 (10.3)	1.1 (0.6-2.3)	1/29 (3.4)	2.7 (0.3-23.5)	12/29 (41.4)	6.0 (3.7-9.7
All preterm newborns	127/780 (16.3)	1.7 (1.4-2.0)	115/780 (14.8)	1.4 (1.1-1.7)	23/780 (3.0)	2.5 (1.3-4.7)	91/780 (11.7)	1.1 (0.9-1.4)
Term newborn group	356/3224 (11.0)	1 [Reference]	326/3224 (10.1)	1 [Reference]	37/3234 (1.1)	1 [Reference]	315/3224 (9.8)	1 [Reference]

Abbreviations: NA, not applicable; OR, odds ratio.

^a^ Adjusted for study site. 95% CIs are based on robust SEs.

^b^ Infections include treatment with antibiotic medication (≥3 regimens), otitis media, pneumonia, bronchiolitis, parasitosis, diarrhea, vomiting, exanthema, skin disease, fever lasting 3 days or more (≥3 episodes), meningitis, and other infections that required antibiotic treatment.

^c^ Neurologic disorders include seizures, cerebral palsy, and other neurologic disorders.

^d^ Severe clinical condition includes cardiovascular problems, gastroesophageal reflux, any hemolytic condition, injury trauma, and any condition that required surgery.

### Neurodevelopmental Outcomes

**Figure 3.**
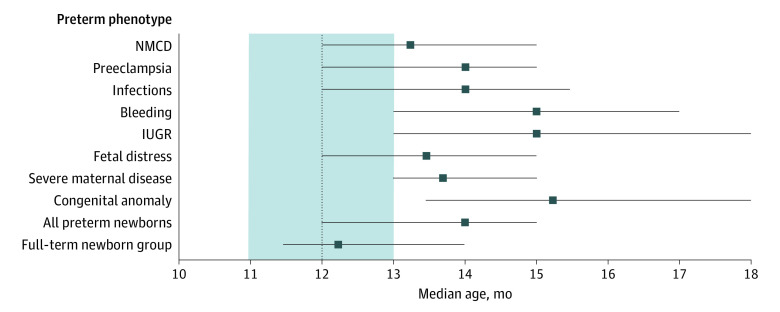
Median Age for Developmental Achievement of Walking Alone According to Preterm-Birth Phenotype Horizontal lines represent interquartile ranges. For comparison, the 25th and 75th centiles of the WHO periods of achievement for the same developmental milestone^29^ are shown in the shaded area (with the median age represented by a broken vertical line). IUGR indicates intrauterine growth restriction; NMCD, no main condition detected; and WHO, World Health Organization.

Figure 3 presents the median age (with 25th-75th centiles) of achievement for the WHO gross motor development milestone of age at walking alone because this milestone is the least subject to maternal recall bias and because our data could be compared with the age ranges for achievement of WHO milestones.^29^ Newborns with the phenotypes for intrauterine growth restriction, bleeding, and congenital anomaly were considerably slower in reaching this milestone, with median ages at achievement of 15.0 months (25th-75th centile, 13.0-18.0 months), 15.0 months (25th-75th centile, 13.0-17.0 months), and 15.2 months (25th-75th centile, 13.5-18.0 months), respectively, representing a 3-month delay compared with the median age of 12 months reported by the WHO.^29^ Newborns with other phenotypes experienced a 1- to 2-month delay.

Table 2 presents the risk of scoring lower than the 10th centile of the normative population for the INTER-NDA domains^24^ according to phenotype and compared with the term newborn group. Heterogeneity was observed, which would have been missed if all preterm newborns had been considered as a single entity. For example, excluding newborns with the congenital anomaly phenotype, those with the fetal distress phenotype had the highest risk of cognitive (OR, 5.1; 95% CI, 2.3-11.1), fine motor (OR, 10.6; 95% CI, 5.1-22.2), gross motor (OR, 3.9; 95% CI, 1.8-8.5), and language (OR, 6.3; 95% CI, 3.2-12.2) development problems. Newborns with the preeclampsia phenotype had a high risk of cognitive (OR, 2.7; 95% CI, 1.4-5.0), fine motor (OR, 4.7; 95% CI, 1.2-18.3), and gross motor (OR, 4.1; 95% CI, 2.4-7.0) development problems. Compared with term newborns, the highest risk of scoring lower than the 10th centile based on INTER-NDA normative values was observed in the fine motor development domain among newborns with the fetal distress (OR, 10.6; 95% CI, 5.1-22.2) and congenital anomaly (OR, 31.1; 95% CI, 3.2-300.3) phenotypes. The risk of fine motor delay was high among all preterm-birth phenotypes.

**Table 2.** Risk of Scoring Lower Than the 10th Centile on the INTERGROWTH-21st Neurodevelopment Assessment at Age 2 Years According to Preterm-Birth Phenotype^**a**^

Variable	Infants, No.	OR (95% CI)					
		Cognitive development	Motor development		Language development	Behavior	
			Fine	Gross		Positive	Negative^b^
Phenotype							
No main condition detected	252	2.0 (1.6-2.5)	NA	1.2 (0.4-3.4)	1.6 (0.8-3.2)	1.3 (1.1-1.7)	1.3 (1.1-1.6)
Preeclampsia	84	2.7 (1.4-5.0)	4.7 (1.2-18.3)	4.1 (2.4-7.0)	1.2 (0.8-1.8)	1.4 (0.9-2.2)	2.0 (1.3-3.1)
Infections	129	1.6 (0.7-3.4)	5.1 (1.7-15.4)	2.6 (1.4-4.9)	2.0 (0.8-5.1)	1.1 (0.8-1.7)	1.5 (1.3-1.7)
Bleeding	42	0.5 (0.1-2.9)	NA	2.9 (1.2-7.2)	NA	0.1 (0.0-0.6)	0.6 (0.2-1.6)
Intrauterine growth restriction	45	0.5 (0.1-4.9)	NA	2.0 (1.3-3.1)	1.2 (0.4-3.8)	1.5 (0.9-2.5)	1.4 (0.8-2.3)
Fetal distress	67	5.1 (2.3-11.1)	10.6 (5.1-22.2)	3.9 (1.8-8.5)	6.3 (3.2-12.2)	1.7 (0.7-3.7)	1.6 (0.9-2.8)
Severe maternal disease	51	2.1 (1.0-4.4)	3.9 (2.8-5.6)	0.6 (0.2-1.9)	2.4 (1.0-5.8)	1.9 (1.1-3.5)	1.3 (0.5-3.5)
Congenital anomaly	27	5.6 (1.9-16.2)	31.1 (3.2-300.3)	6.6 (0.8-57.2)	7.2 (0.6-86.8)	2.8 (1.0-8.2)	3.0 (1.3-6.6)
All preterm newborns	697	2.2 (1.6-3.0)	4.2 (1.7-9.9)	2.2 (1.4-3.5)	2.1 (1.3-3.3)	1.3 (1.2-1.5)	1.4 (1.3-1.6)
Term newborn group	2965	1 [Reference]	1 [Reference]	1 [Reference]	1 [Reference]	1 [Reference]	1 [Reference]

Abbreviations: INTERGROWTH-21st, International Fetal and Newborn Growth Consortium for the 21st Century; NA, not applicable; OR, odds ratio.

^a^ Adjusted for study site. 95% CIs are based on robust SEs.

^b^ Negative behavior is defined as a risk of negative behavior that scored higher than the 90th centile on the INTERGROWTH-21st Neurodevelopment Assessment at age 2 years according to preterm-birth phenotype compared with the term newborn group.

## Discussion

The study’s findings indicated that, among an international sample selected to maximize the inclusion of high-risk pregnancies, differential patterns existed with regard to maternal risk factors, the incidence of newborns who were small for gestational age, neonatal and childhood growth, neonatal and childhood severe morbidity, and neurodevelopment across preterm-birth phenotypes. These differential patterns are likely associated with specific pathologic factors that have implications for the growing fetus, as previously reported for early neonatal outcomes.^5^

The postnatal patterns varied across phenotype and age. For example, newborns with the intrauterine growth restriction phenotype had lower neonatal morbidity but more growth restriction and neurodevelopmental delay in childhood. Newborns with the no main condition phenotype who were not small for their gestational age had lower neonatal morbidity but increased morbidity at age 2 years. Those with the infections phenotype had lower gestational ages at birth and a low risk of being small for gestational age but a high risk of morbidity and postnatal growth restriction.

The association between preterm birth and the phenotype for intrauterine growth restriction, which was observed in 8.0% of all preterm infants, would not have emerged had the syndrome’s heterogeneity not been recognized. Notably, some preterm newborns with the intrauterine growth restriction phenotype were not included in that phenotype group for this study because they had other pathologic characteristics, such as preeclampsia or congenital anomalies. Other etiologic factors were likely associated with fetal growth restriction (eFigure 2 in the Supplement).^1^

Our results support clustering 1 or more maternal, fetal, or placental conditions to construct phenotypes rather than relying on 1 associated factor. However, 35.1% of preterm births were clustered in the no main condition phenotype, which likely reflects reliance on the use of clinical parameters alone for classification or suggests more complex underlying associations between environmental and nutritional factors.

In this study, the dominant pathologic characteristics in the remaining phenotypes (3 maternal, 3 fetal, and 1 placental)^2^ commonly occurred with complications and comorbidities (particularly extrauterine infection, chorioamnionitis, and perinatal sepsis) that were associated with the dominant condition and preterm birth (eTable 3 in the Supplement). Nevertheless, there are sufficient data to justify targeting the main putative conditions for the prevention and clinical management of these preterm-birth phenotypes.

The present study is distinct because (1) it has a firm conceptual basis^2,3,4^; (2) it was designed to explore a priori hypotheses associated with the heterogeneity of preterm birth; (3) it accurately estimated gestational age in all newborns; (4) it oversampled newborns with low gestational ages to maximize the number of subgroups considered very high risk; (5) it achieved a 1:4 ratio for enrolled preterm and term newborns, substantially exceeding the 1:1 target; (6) it monitored growth, morbidity, and neurodevelopment from birth to age 2 years using standardized data collection systems; and (7) it assessed neurodevelopment compared with international normative values using a validated psychometric tool.^24,30^

The study’s results have implications for research and clinical practice. Preterm birth is a distinct syndrome in the medical field because it is defined by time (ie, gestational age at birth rather than etiologic, clinical, nutritional, or laboratory characteristics, as with other syndromes). Hence, it is important to estimate the gestational age as accurately as possible, consistent with the 2016 WHO guidelines,^31^ and to avoid the use of low birth weight (ie, <2500 g regardless of gestational age) as a factor.^32^ It has been asserted that low birth weight is a necessary factor to include because, in many regions of the world, gestational age cannot be reliably estimated. However, actions to minimize the incidence of preterm birth may nonetheless be taken and are warranted for a syndrome that occurs in at least 10% to 12% of all births worldwide and has serious implications for human developmental capacity. Governments, international organizations, and donors can work to improve antenatal care so that every woman receives adequate evaluation early in pregnancy.^31^

### Limitations

This study has several limitations. The loss to follow-up at age 2 years was higher than that of previous studies,^30,33^ as some sites were located in rural or semiurban areas. This limitation was considered when the INTERBIO-21st study was designed, but we concluded that the loss of follow-up at such sites was offset by the enrollment of a high-risk population with accurate gestational ages and a higher number of more severe exposures (eg, infections and inadequate nutrition). Nevertheless, the risk of systematic loss to follow-up bias according to phenotype is unlikely because, with the exception of the intrauterine growth restriction phenotype, the follow-up rates across phenotypes were similar.

We could not include multiple births because we focused, for several logistical and sample size reasons, on singletons. In addition, we did not include stillbirths or late terminations because the childhood follow-up component was fundamental to the study. We also did not document the ways in which urinary tract infections were specifically diagnosed or record whether prophylactic antibiotic medications were prescribed for the treatment of group B streptococcus colonization. We categorized congenital anomaly as a separate phenotype to reinforce the integrity of preterm birth as a distinct syndrome. However, given the small sample of infants with the congenital anomaly phenotype (3.5% of all preterm newborns), we could not draw meaningful conclusions.

Although cluster methods are susceptible to nuances in the data, the fact that we obtained clusters that were similar to those acquired in a previous study,^5^ using identical methods but a data set with a different risk profile, supports our hypothesis that these phenotypes occur systematically across populations. However, the phenotype prevalence changes depending on which factors predominate.

We recognize that there are competing etiologic characteristics and risk factors among the preterm birth, intrauterine growth restriction, and stillbirth syndromes,^34^ which were highlighted by Lee et al.^35^ Our study was designed to explore the preterm and intrauterine growth restriction syndromes in parallel. This overlapping approach was required because intrauterine growth restriction is an equally complex syndrome that has multiple etiologic characteristics and presentations. However, our focus on developmental outcomes precluded including stillbirths. Future research may explore these associations in detail.

## Conclusions

In this study, the preterm birth syndrome was composed of well-defined phenotypes with differential neonatal morbidity, early childhood morbidity, growth, and neurodevelopment up to age 2 years. Therefore, the concept of preterm birth as an exclusively time-based entity may no longer be appropriate. Phenotypic classification of preterm newborns is likely to provide a better understanding of the etiologic factors and mechanisms associated with preterm birth. Although the study’s findings indicated that approximately 35% of phenotypes were not associated with distinct clinical conditions, newborns with preterm-birth phenotypes nevertheless remain at a higher risk of growth and developmental problems.

## Notes

Supplement. **eTable 1.** Definitions of Preterm-Birth Phenotypes **eTable 2.** Baseline Characteristics and Perinatal Events According to Preterm-Birth Phenotypes **eTable 3.** Distribution of Preterm-Birth Phenotypes According to the Main Maternal, Fetal, or Placental Conditions **eTable 4.** Median, 25th Centile, and 75th Centile *z* Scores at Birth and Ages 1 and 2 Years According to Preterm-Birth Phenotype **eTable 5.** Morbidity of Children Over the First Year of Age According to Preterm-Birth Phenotype Compared With Term Newborn Group **eFigure 1.** Study Flowchart of the INTERBIO-21st Newborn Study **eFigure 2.** Risk Factors According to Preterm-Birth Phenotype: Adjusted Odds Ratios and 95% CIs for Preterm-Birth Phenotypes Compared With Term Newborn Group **eFigure 3.** Birth Weight Lower Than the 10th Centile of the International INTERGROWTH-21st Newborn Size Standards and Very Preterm Size on Birth Reference Charts According to Preterm-Birth Phenotype
