# Supplementary material for: Association Between Preterm-Birth Phenotypes and Differential Morbidity, Growth, and Neurodevelopment at Age 2 Years: Results From the INTERBIO-21st Newborn Study
Source: JAMA Pediatr. 2021 Mar 1;175(5):1–11. doi: 10.1001/jamapediatrics.2020.6087 (PMC7922239; doi:10.1001/jamapediatrics.2020.6087)
Supplement: Supplement. — eTable 1. Definitions of Preterm-Birth Phenotypes eTable 2. Baseline Characteristics and Perinatal Events According to Preterm-Birth Phenotypes eTable 3. Distribution of Preterm-Birth Phenotypes According to the Main Maternal, Fetal, or Placental Conditions eTable 4. Median, 25th Centile, and 75th Centile z Scores at Birth and Ages 1 and 2 Years According to Preterm-Birth Phenotype eTable 5. Morbidity of Children Over the First Year of Age According to Preterm-Birth Phenotype Compared With Term Newborn Group eFigure 1. Study Flowchart of the INTERBIO-21st Newborn Study eFigure 2. Risk Factors According to Preterm-Birth Phenotype: Adjusted Odds Ratios and 95% CIs for Preterm-Birth Phenotypes Compared With Term Newborn Group eFigure 3. Birth Weight Lower Than the 10th Centile of the International INTERGROWTH-21st Newborn Size Standards and Very Preterm Size on Birth Reference Charts According to Preterm-Birth Phenotype [file jamapediatr-e206087-s001.pdf]

Supplementary Online Content

Villar J, Restrepo-Méndez MC, McGready R, et al. Association between preterm-birth phenotypes and differential morbidity, growth, and neurodevelopment at age 2 years: results from the INTERBIO-21st Newborn Study. *JAMA Pediatr.* Published online March 1, 2021. doi:10.1001/jamapediatrics.2020.6087

eTable 1. Definitions of Preterm-Birth Phenotypes

eTable 2. Baseline Characteristics and Perinatal Events According to Preterm-Birth Phenotypes

eTable 3. Distribution of Preterm-Birth Phenotypes According to the Main Maternal, Fetal, or Placental Conditions

eTable 4. Median, 25th Centile, and 75th Centile *z* Scores at Birth and Ages 1 and 2 Years According to Preterm-Birth Phenotype

eTable 5. Morbidity of Children Over the First Year of Age According to Preterm-Birth Phenotype Compared With Term Newborn Group

eFigure 1. Study Flowchart of the INTERBIO-21st Newborn Study

eFigure 2. Risk Factors According to Preterm-Birth Phenotype: Adjusted Odds Ratios and 95% CIs for Preterm-Birth Phenotypes Compared With Term Newborn Group

eFigure 3. Birth Weight Lower Than the 10th Centile of the International INTERGROWTH-21st Newborn Size Standards and Very Preterm Size on Birth Reference Charts According to Preterm-Birth Phenotype

This supplementary material has been provided by the authors to give readers additional information about their work.

© 2021 Villar J et al. *JAMA Pediatrics*.

eTable 1. Definitions of Preterm-Birth Phenotypes

Preterm phenotypes	Conditions	Definition
No main condition detected	No conditions detected	No main maternal, fetal or placental condition detected.
Mostly preeclampsia	Preeclampsia	Defined as elevated blood pressure (≥140/90 mm Hg), 30 mm Hg increase of systolic pressure, or 15 mm Hg increase of diastolic pressure in relation to basal measurements observed at least twice, the interval of the measurements being >4 h but <168 h and proteinuria >2+ by dipstick.
	Eclampsia	Defined as the occurrence of seizures (grand mal type) and/or coma, not related to cerebral problems, in women who presented with signs of preeclampsia. Symptoms might have occurred before or during labor or within 48 h after delivery.
	HELLP syndrome	HELLP or any other coagulation abnormalities reported from a pregnant woman with preeclampsia or eclampsia.
	Third trimester bleeding and preeclampsia	Vaginal bleeding occurring $>27^{+0}$ weeks' gestation in women diagnosed as having severe preeclampsia, eclampsia or HELLP syndrome.
Mostly infection-related	Extrauterine infection during the index pregnancy	Presence of at least one of the following: malaria, pyelonephritis, sexually transmitted diseases (including syphilis and HIV/AIDS), and other clinically documented infections that required use of antibiotics or other treatments during pregnancy, except when antibiotics were used for PPROM.
	Clinical chorioamnionitis	Cases where antibiotic treatment was specifically indicated for PPROM or diagnosed/treated cases reported for a woman or documented in the medical records. Suspected chorioamnionitis cases with intact membranes were not possible to identify in this data set.
	Fetal inflammatory response syndrome or perinatal sepsis	Signs, symptoms, and laboratory results compatible with perinatal sepsis documented by the neonatologist (systemic illness with bacteremia).
	Fetal anemia	For example, due to fetal hemolytic disease; Rhesus negative.

© 2021 Villar J et al. *JAMA Pediatrics*.

Preterm phenotypes	Conditions	Definition
Mostly bleeding-related	Early bleeding	Vaginal bleeding <15^{+0} weeks' gestation.
	Mid/late-pregnancy bleeding	Vaginal bleeding ≥15^{+0} weeks' gestation without the diagnosis of preeclampsia, eclampsia or HELLP syndrome.
Mostly suspected IUGR	Suspicion or diagnosis of intrauterine growth restriction	Suspicion of impaired fetal growth during pregnancy based on ultrasound examinations or physical examination, and specifically stated in the medical record
Mostly fetal distress	Fetal distress	Diagnosis based on: (1) abnormal antepartum nonstress test reported in the medical record as indication for induction of labor or elective Cesarean delivery or (2) severe intrapartum electronic fetal monitoring pattern equivalent to category 3 of NICHD as indication for intrapartum Cesarean delivery
Mostly severe maternal condition	Severe maternal disease clinically active during the index pregnancy	Cases with a relevant clinical condition documented in the medical records in which birth was caregiver initiated because of the severity or complications related to these conditions. This excludes cases in which there was also an obstetric reason for induction/Cesarean delivery. Clinical conditions associated with caregiver-initiated preterm birth included diabetes mellitus, thyroid disease, other endocrine diseases, cardiac disease, hypertension previous to pregnancy, chronic respiratory disease (including chronic asthma), renal disease, cancer, lupus erythematosus, any coagulopathy (including falciparum anemia), tuberculosis, severe intestinal malabsorption (including Crohn's and celiac diseases), maternal congenital abnormality or genetic disease (eg, cystic fibrosis or cardiac congenital defects), epilepsy, or any other clinical condition that required surgery or referral to specialized care
Mostly congenital anomaly	Fetal anomaly	Severe anomalies diagnosed through ultrasound in pregnancy or on neonatal examination

Notes:
HELLP: hemolysis, elevated liver enzymes, low platelet
HIV: human immunodeficiency virus
NICHD: National Institute of Child Health and Human Development
PPROM: preterm premature rupture of membranes.

© 2021 Villar J et al. *JAMA Pediatrics*.

eTable 2. Baseline Characteristics and Perinatal Events According to Preterm-Birth Phenotypes

	**NMCD***		**Preeclampsia***		**Infections***		**Bleeding***		**IUGR***		**Fetal distress***		**Severe maternal disease***		**Congenital anomaly***		**All preterm newborns***		**Term newborn group***	
	n=485		n=162		n=289		n=71		n=110		n=131		n=85		n=48		n = 1381		n = 5148	
Maternal age, years	28.7	0.3	29.8	0.5	29.0	0.4	29.8	0.6	28.8	0.5	30.0	0.5	30.3	0.7	30.7	0.9	29.3	0.2	29.3	0.1
Maternal height, cm	158.1	0.4	160.5	0.5	158.7	0.5	162.4	0.9	159.5	0.7	160.5	0.6	160.9	0.9	159.2	0.9	159.3	0.2	159.8	0.1
Maternal weight, kg	62.6	0.7	71.8	1.4	62.1	0.9	66.9	1.8	63.9	1.4	69.2	1.4	66.8	1.8	62.1	2.3	64.8	0.4	63.9	0.2
Body-mass index, kg/m2	25.0	0.2	27.8	0.5	24.5	0.3	25.2	0.6	25.1	0.5	26.8	0.5	25.8	0.6	24.4	0.8	25.4	0.1	24.9	0.1
Gestational age at first visit, weeks	14.4	0.2	13.9	0.3	13.9	0.3	13.7	0.4	13.3	0.3	13.6	0.3	13.2	0.4	13.9	0.6	14.0	0.1	13.4	0.1
Gestational age at birth, weeks	34.9	0.1	34.0	0.2	33.9	0.2	33.8	0.3	34.8	0.2	33.9	0.2	34.8	0.2	33.9	0.4	34.4	0.1	39.4	0.0
Years of formal education, years	10.4	0.3	12.5	0.4	11.1	0.3	13.1	0.5	13.8	0.4	12.9	0.3	12.8	0.5	10.5	0.9	11.6	0.1	11.8	0.1
Married or cohabiting	43	9%	9	6%	36	12%	5	7%	12	11%	27	21%	11	13%	6	13%	149	11%	489	9%
Nulliparous	202	42%	79	49%	131	45%	22	31%	51	46%	65	50%	38	45%	20	42%	608	44%	2049	40%
Pre-eclampsia	0	0%	162	100%	6	2%	0	0%	21	19%	28	21%	0	0%	5	10%	222	16%	132	3%
Pyelonephritis	0	0%	3	2%	12	4%	0	0%	1	1%	3	2%	0	0%	1	2%	20	1%	44	1%
Any sexually transmitted infection	7	1%	2	1%	5	2%	2	3%	1	1%	0	0.0%	5	6%	0	0.0%	22	2%	119	2%

*** Data are mean (SD) or number (%).** IUGR: intrauterine growth restriction; NMCD: no main condition detected.

© 2021 Villar J et al. *JAMA Pediatrics*.

	NMCD*		Preeclampsia*		Infections*		Bleeding*		IUGR*		Fetal distress*		Severe maternal disease*		Congenital anomaly*		All preterm newborns*		Term newborn group*	
	n=485		n=162		n=289		n=71		n=110		n=131		n=85		n=48		n = 1381		n = 5148	
Spontaneous initiation of labour	363	75%	17	10%	188	65%	18	25%	9	8%	38	29%	18	21%	30	63%	681	49%	3179	62%
PPROM	131	27%	11	7%	174	60%	14	20%	8	7%	30	23%	32	38%	17	35%	417	30%	413	8%
Caesarean section	163	34%	124	77%	78	27%	61	86%	76	69%	123	94%	55	65%	16	33%	696	50%	1778	35%
NICU admission >1 day	185	38%	101	62%	200	69%	51	72%	63	57%	86	66%	38	45%	31	65%	755	55%	380	7%
LBW (<2500g)	285	59%	115	71%	205	71%	50	70%	107	97%	100	76%	48	56%	37	77%	947	69%	247	5%
Neonatal mortality	17	4%	14	9%	8	3%	4	6%	1	1%	5	4%	2	2%	4	8%	55	4%	8	0.2%
Male sex	247	51%	69	43%	151	52%	42	59%	54	49%	65	50%	47	55%	28	58%	703	51%	2609	51%
Exclusive breastfeeding at discharge	381	79%	89	55%	173	60%	32	45%	48	44%	75	57%	50	59%	18	38%	866	63%	4439	86%
Mother admitted to ICU	4	1%	6	4%	1	0%	1	1%	1	1%	2	2%	2	2%	0	0%	17	1%	21	0.4%
Birth weight, g	2363	23.9	2099	54.1	2186	33.0	2198	63.8	1831	36.0	2079	62.6	2358	56.4	2152	96.5	2210	15.9	3200	6.2
Birth length, cm	45.4	0.1	44.4	0.3	44.5	0.2	44.4	0.4	42.7	0.3	43.8	0.4	45.3	0.3	43.8	0.5	44.6	0.1	48.9	0.0
Birth head circumference, cm	31.7	0.1	31.3	0.2	31.1	0.1	30.9	0.3	30.2	0.2	30.8	0.3	31.8	0.2	31.0	0.4	31.3	0.1	33.9	0.0

*** Data are mean (SD) or number (%).** IUGR: intrauterine growth restriction; NMCD: no main condition detected. PPROM=preterm prelabor rupture of membranes; NICU=neonatal intensive care unit; LBW=low birthweight; ICU=intensive care unit.

© 2021 Villar J et al. *JAMA Pediatrics*.

eTable 3. Distribution of Preterm-Birth Phenotypes According to the Main Maternal, Fetal, or Placental Conditions

**Preterm phenotype** (Main condition)	**Preterm newborns <37^{+0} weeks' gestation**			
	**N**	**%**	**% Main condition**	**Most frequent associated conditions (%)**
**No main condition detected** (NMCD)	485	35	0	None
**Preeclampsia** (Mostly preeclampsia)	162	12	100	Perinatal sepsis (16)<br>Extrauterine infection (11)<br>Suspected IUGR (9)
**Infections** (Mostly infection-relatedǂ)	289	21	100	Chorioamnionitis (48)<br>Extrauterine infection (39)<br>Perinatal sepsis (35)
**Bleeding** (Mostly bleeding-related ǂǂ)	71	5	100	Severe maternal condition (30)<br>Perinatal sepsis (24)<br>Chorioamnionitis (10)<br>Extrauterine infection (9)
**IUGR** (Mostly suspected IUGR)	110	8	100	Severe maternal condition (34)<br>Preeclampsia (19)<br>Perinatal sepsis (10)<br>Extrauterine infection (8)<br>Mid-late pregnancy bleeding (8)
**Fetal distress** (Mostly fetal distress)	131	9	100	Preeclampsia (21)<br>Extrauterine infection (21)<br>Suspected IUGR (20)<br>Severe maternal condition (18)<br>Perinatal sepsis (15)<br>Mid-late pregnancy bleeding (12)
**Severe maternal disease** (Mostly severe maternal disease)	85	6	66	Early bleeding (45)<br>Extrauterine infection (24)<br>Mid-late pregnancy bleeding (11)<br>Suspected IUGR (8)
**Congenital anomaly** (Mostly congenital anomaly)	48	4	100	Perinatal sepsis (31)<br>Chorioamnionitis (25)<br>Extrauterine infection (23)<br>Suspected IUGR (13)<br>Early bleeding (13)
Total	1381	100		

ǂ This preterm phenotype was generated by merging extrauterine infection, chorioamnionitis and perinatal sepsis phenotypic clusters. In addition, fetal anaemia cases were included in this group as the majority of cases (64%) were associated with perinatal sepsis.
ǂǂ This preterm phenotype was generated by merging mid-late pregnancy bleeding (without preeclampsia; hemolysis, elevated liver enzymes and low platelet count (HELLP) or eclampsia) and early bleeding phenotype clusters.
IUGR: Intrauterine growth restriction. Main condition: Predominant condition within each cluster.

© 2021 Villar J et al. *JAMA Pediatrics*.

eTable 4. Median, 25th Centile, and 75th Centile *z* Scores at Birth and Ages 1 and 2 Years According to Preterm-Birth Phenotype

**At birth**		**Weight**			**Length**			**Head circumference**			**Weight-for-length**		
	**N**	**Median**	**25th**	**75th**	**Median**	**25th**	**75th**	**Median**	**25th**	**75th**	**Median**	**25th**	**75th**
NMCD	485	-0.2	-0.8	0.4	-0.3	-0.9	0.5	-0.1	-0.8	0.6	-0.2	-0.7	0.3
Pre-eclampsia	162	-0.7	-1.3	0.4	-0.4	-1.1	0.3	-0.2	-1.0	0.7	-0.5	-1.3	0.4
Infections	289	-0.1	-0.7	0.5	-0.2	-0.9	0.3	-0.2	-0.7	0.7	-0.2	-0.7	0.4
Bleeding	71	-0.1	-0.6	0.6	-0.2	-0.7	0.5	-0.1	-0.5	0.5	-0.2	-0.5	0.5
IUGR	110	-1.6	-2.1	-1.1	-1.7	-2.4	-1.0	-1.1	-1.9	-0.7	-1.6	-2.1	-1.0
Fetal distress	131	-0.4	-1.4	0.3	-0.6	-1.3	0.2	-0.4	-1.2	0.5	-0.4	-1.3	0.2
Severe maternal disease	84	-0.1	-0.5	0.5	-0.2	-0.9	0.5	0.1	-0.8	0.9	-0.1	-0.5	0.4
Congenital anomaly	48	0.0	-0.7	0.5	-0.3	-1.1	0.2	-0.2	-1.1	0.5	0.0	-0.8	0.5
All preterm newborns	1380	-0.3	-1.0	0.4	-0.4	-1.1	0.3	-0.2	-1.0	0.5	-0.3	-0.9	0.3
Term newborn group	5148	-0.2	-0.8	0.5	-0.2	-0.9	0.5	0.1	-0.6	0.8	-0.2	-0.8	0.5

NMCD: no main condition detected
IUGR: intrauterine growth restriction

**At 1 year**		**Weight**			**Length**			**Head circumference**			**Weight-for-length**		
	**N**	**Median**	**25th**	**75th**	**Median**	**25th**	**75th**	**Median**	**25th**	**75th**	**Median**	**25th**	**75th**
NMCD	286	-0.3	-1.3	0.4	-1.0	-2.0	-0.1	-0.4	-1.5	0.5	0.1	-0.7	0.9
Pre-eclampsia	95	0.0	-0.9	0.5	-0.7	-1.6	-0.1	0.0	-0.8	0.7	0.4	-0.4	1.0
Infections	139	-0.4	-1.3	0.4	-1.1	-1.9	-0.3	-0.4	-1.2	0.5	0.1	-0.5	0.9
Bleeding	40	-0.3	-0.9	0.4	-0.6	-1.2	0.3	-0.2	-1.2	0.6	0.1	-0.5	0.8
IUGR	48	-1.1	-1.8	-0.2	-1.3	-2.1	-0.9	-1.2	-1.8	-0.4	-0.7	-1.5	0.4
Fetal distress	72	-0.3	-1.1	0.5	-1.5	-2.2	-0.3	0.0	-1.1	0.4	0.4	-0.6	1.3
Severe maternal disease	48	0.2	-0.6	0.8	-0.5	-1.4	0.1	0.0	-0.7	0.7	0.5	-0.2	0.9
Congenital anomaly	19	-0.2	-2.0	0.5	-1.2	-1.9	-0.8	-0.8	-2.4	0.2	-0.2	-1.3	1.2
All preterm newborns	747	-0.3	-1.2	0.4	-1.0	-1.9	-0.2	-0.3	-1.2	0.5	0.2	-0.7	0.9
Term newborn group	3372	0.0	-0.8	0.8	-0.4	-1.2	0.4	0.0	-0.8	0.8	0.3	-0.5	1.1

NMCD: no main condition detected
IUGR: intrauterine growth restriction

© 2021 Villar J et al. *JAMA Pediatrics*.

At 2 years		Weight			Length			Head circumference			Weight-for-length		
	N	Median	25th	75th	Median	25th	75th	Median	25th	75th	Median	25th	75th
NMCD	280	-0.4	-1.4	0.6	-0.6	-1.6	0.3	-0.2	-1.3	0.6	0.0	-0.9	0.9
Pre-eclampsia	92	-0.1	-0.8	0.7	-0.3	-1.1	0.4	0.2	-0.6	0.8	0.1	-0.7	1.0
Infections	146	-0.3	-1.0	0.5	-0.8	-1.7	0.1	-0.3	-1.2	0.6	0.1	-0.6	0.7
Bleeding	44	0.0	-0.6	0.7	-0.3	-0.8	0.6	-0.4	-1.2	0.7	0.1	-0.4	0.8
IUGR	46	-0.9	-1.7	-0.1	-0.9	-1.7	-0.5	-1.0	-1.5	-0.2	-0.4	-1.3	0.2
Fetal distress	73	-0.3	-1.2	0.6	-0.7	-1.5	0.3	-0.2	-1.2	0.9	0.1	-0.8	1.0
Severe maternal disease	54	0.1	-0.5	1.1	-0.2	-1.1	0.6	-0.3	-0.8	0.6	0.2	-0.4	0.8
Congenital anomaly	29	-0.5	-1.7	0.9	-1.2	-1.7	0.2	-1.0	-1.8	0.3	-0.2	-1.2	1.4
All preterm newborns	764	-0.3	-1.1	0.6	-0.6	-1.5	0.3	-0.2	-1.2	0.6	0.1	-0.8	0.8
Term newborn group	3157	0.0	-0.8	0.8	-0.3	-1.1	0.6	0.0	-0.9	0.8	0.1	-0.6	0.9

NMCD: no main condition detected
IUGR: intrauterine growth restriction

© 2021 Villar J et al. *JAMA Pediatrics*.

eTable 5. Morbidity of Children Over the First Year of Age According to Preterm-Birth Phenotype Compared With Term Newborn Group

		**Hospitalized at least once**				**Infections (at least 3)**				**Neurologic disorders**				**Severe clinical condition**			
	**N**	**OR**	**95% CI**			**OR**	**95% CI**			**OR**	**95% CI**			**OR**	**95% CI**		
NMCD	289	2.2	1.8	-	2.7	1.0	0.6	-	1.5	4.6	2.1	-	9.8	1.2	0.9	-	1.6
Preeclampsia	95	1.8	1.1	-	3.2	1.9	1.5	-	2.6	1.6	0.5	-	5.2	1.1	0.7	-	2.0
Infections	141	3.4	2.3	-	5.2	1.1	0.5	-	2.3	3.4	0.4	-	30.0	1.8	1.3	-	2.4
Bleeding	40	1.7	1.0	-	3.1	1.2	0.4	-	3.8	3.5	1.2	-	10.2	2.3	1.2	-	4.3
IUGR	48	1.5	0.6	-	3.9	2.0	1.2	-	3.3	2.8	1.0	-	7.6	1.6	0.9	-	2.8
Fetal distress	73	3.7	2.4	-	5.9	1.1	0.7	-	1.7	4.4	1.0	-	18.9	2.2	1.7	-	2.8
Severe maternal disease	48	1.2	0.6	-	2.2	0.8	0.6	-	1.1	1.0	--	-	--	1.5	0.7	-	3.0
Congenital anomaly	19	6.9	3.4	-	14.0	2.1	0.8	-	5.6	18.0	3.5	-	91.7	6.5	2.8	-	15.4
All preterm newborns	753	2.4	1.9	-	3.0	1.2	0.9	-	1.7	3.7	1.8	-	7.7	1.6	1.2	-	2.1
Term newborn group	3397	1.0	[Reference]			1.0	[Reference]			1.0	[Reference]			1.0	[Reference]		

OR: Odds ratio; 95CI%: 95% confidence interval based on robust standard errors. Adjusted for study site.

NMCD: No main condition detected; IUGR: intrauterine growth restriction

Infections include any of the following conditions: Antibiotics (≥ 3 regimens), otitis media, pneumonia, bronchiolitis, parasitosis, diarrhea, vomiting, exanthema, skin disease, fever ≥3 days (≥3 episodes), meningitis, other infections that required antibiotics

Neurologic disorders include any of the following conditions: Seizures, cerebral palsy, other neurologic disorders

Severe clinical condition includes any of the following conditions: Cardiovascular problems, gastroesophageal reflux, any hemolytic condition, injury trauma, any condition that required surgery

© 2021 Villar J et al. *JAMA Pediatrics*.

eFigure 1. Study Flowchart of the INTERBIO-21st Newborn Study

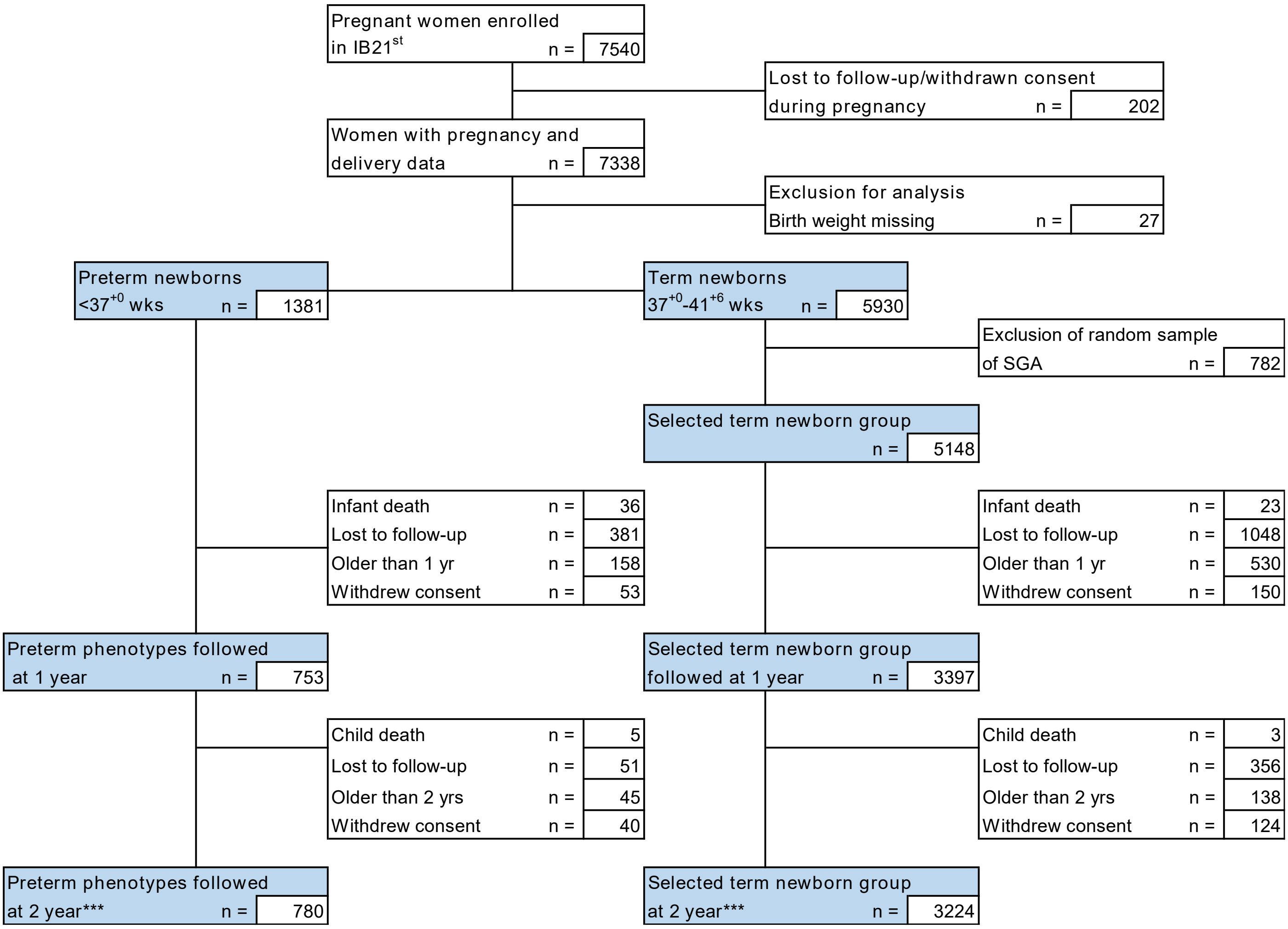

IB21st: INTERBIO-21st Study; SGA: Small-for-gestational-age

*** At 2 years of age, we followed up 168 children born preterm and 448 born term that were not seen at 1 year of age.

© 2021 Villar J et al. *JAMA Pediatrics*.

eFigure 2. Risk Factors According to Preterm-Birth Phenotype: Adjusted Odds Ratios and 95% CIs for Preterm-Birth Phenotypes Compared With Term Newborn Group

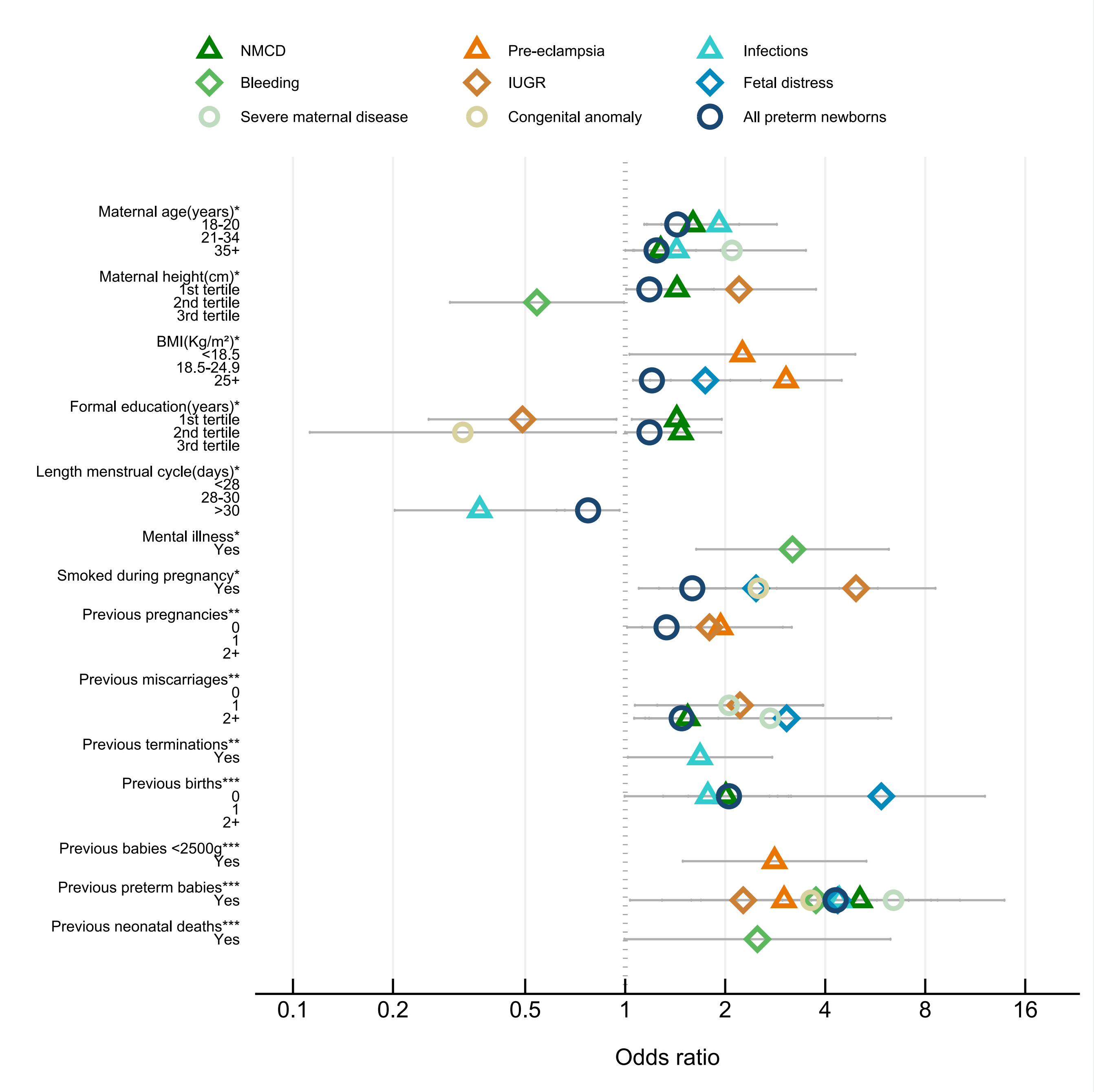

X-axis is in logarithm scale.

IUGR: intrauterine growth restriction; OR: Odds ratio, 95%CI: 95% confidence interval.

Only risk factors with p-values <0.05 and categories which did not include the unit were plotted in the figure.

* Model 1: Adjusted for maternal age, height, first trimester/pre-pregnancy body mass index (BMI), years of education, manual/physically demanding work, pre-pregnancy mental illness, average menstrual cycle length, smoking during pregnancy

** Model 2: adjusted for Model 1 and number of previous pregnancies, miscarriages and terminations

*** Model 3: adjusted for Model 2 and number of previous births, low birth weight (LBW) and preterm babies, and neonatal deaths

© 2021 Villar J et al. *JAMA Pediatrics*.

eFigure 3. Birth Weight Lower Than the 10th Centile of the International INTERGROWTH-21st Newborn Size Standards and Very Preterm Size on Birth Reference Charts According to Preterm-Birth Phenotype

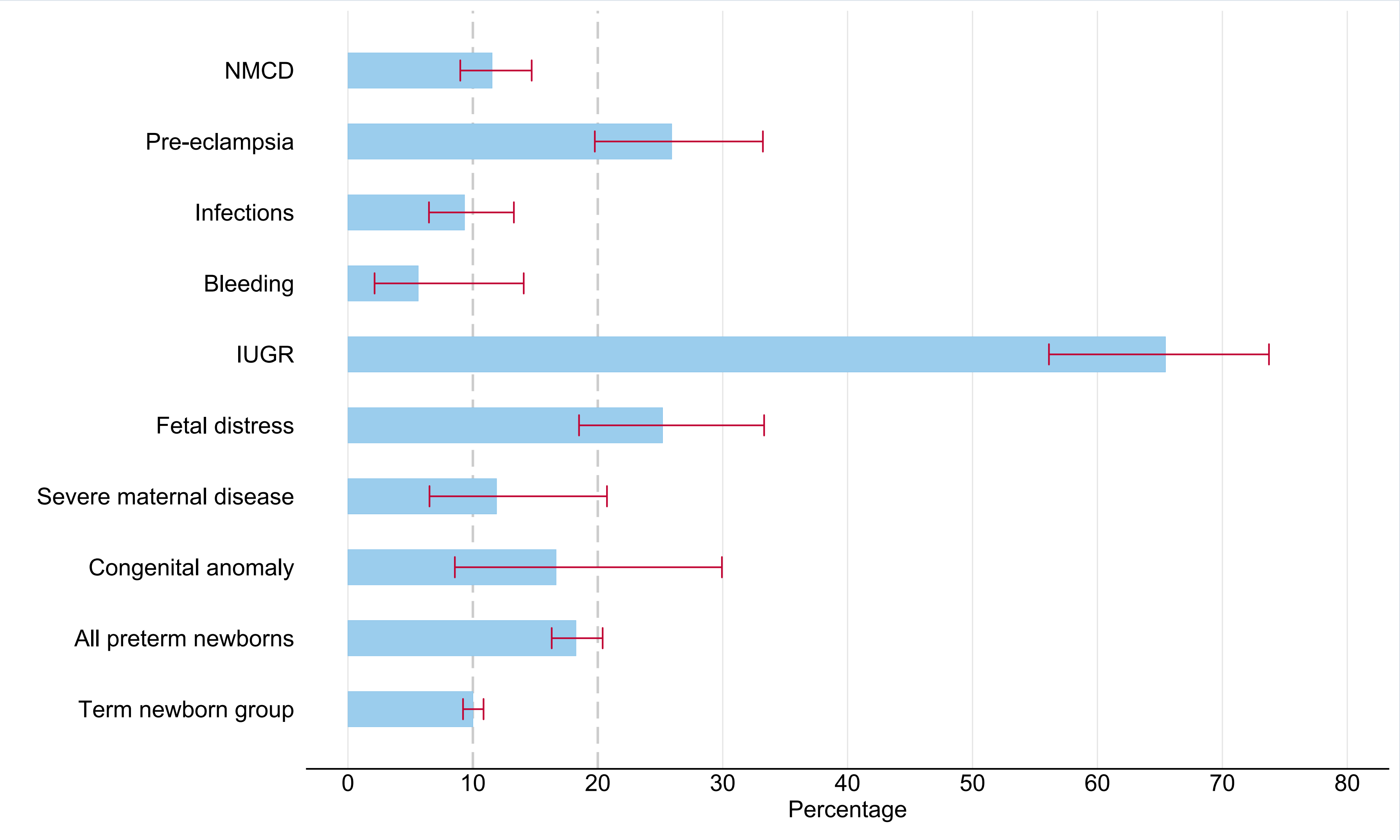

© 2021 Villar J et al. *JAMA Pediatrics*.
